# Expanding Our Understanding of COVID-19 from Biomedical Literature Using Word Embedding

**DOI:** 10.3390/ijerph18063005

**Published:** 2021-03-15

**Authors:** Heyoung Yang, Eunsoo Sohn

**Affiliations:** Future Technology Analysis Center, Korea Institute of Science and Technology Information, 66, Hoegi-ro, Dongdaemun-gu, Seoul 02456, Korea; essohn@kisti.re.kr

**Keywords:** word embedding, machine learning, COVID-19, PubMed literature, drug repurposing, medical subject headings, substance name

## Abstract

A better understanding of the clinical characteristics of coronavirus disease 2019 (COVID-19) is urgently required to address this health crisis. Numerous researchers and pharmaceutical companies are working on developing vaccines and treatments; however, a clear solution has yet to be found. The current study proposes the use of artificial intelligence methods to comprehend biomedical knowledge and infer the characteristics of COVID-19. A biomedical knowledge base was established via FastText, a word embedding technique, using PubMed literature from the past decade. Subsequently, a new knowledge base was created using recently published COVID-19 articles. Using this newly constructed knowledge base from the word embedding model, a list of anti-infective drugs and proteins of either human or coronavirus origin were inferred to be related, because they are located close to COVID-19 on the knowledge base. This study attempted to form a method to quickly infer related information about COVID-19 using the existing knowledge base, before sufficient knowledge about COVID-19 is accumulated. With COVID-19 not completely overcome, machine learning-based research in the PubMed literature will provide a broad guideline for researchers and pharmaceutical companies working on treatments for COVID-19.

## 1. Introduction

Coronavirus disease (COVID-19), caused by severe acute respiratory syndrome coronavirus 2 (SARS-CoV-2), was first identified in Wuhan, China, in December 2019 [1]. The rapid spread of COVID-19 has caused a severe health crisis worldwide and gravely impacted human life and society [2]. The urgent need to develop effective therapeutics and vaccines against COVID-19 is driving numerous clinical studies worldwide. Efforts by several scientists have led to the designing of effective antiviral agents based on an understanding of the SARS-CoV-2′s [3,4] viral genome structure and pathogenicity [5,6], as well as the body’s host response and its protein–protein interactions [7,8,9]. Currently, a few vaccines have been developed. Still safety issues remain in doubt and the supply is insufficient. A therapeutic agent showing a definite effect has not been developed [10].

In addition to clinical-based novel drug development studies, such as antibody therapeutics and plasma therapy, drug repurposing is receiving considerable attention as an alternative for developing COVID-19 treatments [11,12,13]. Several computational drug repurposing studies, including network-based or machine learning-based studies, were conducted to predict drug–target interactions by understanding or utilizing the structural properties of SARS-CoV-2, such as in silico docking and analysis [14], network proximity analysis of drug targets and coronavirus–host interactions in the human interactome [15], and therapeutic target-based virtual ligand screening [16].

Bibliometrics has played a large role as a tool for knowledge discovery. Although traditional bibliometric techniques based on statistics and citation analysis are still widely used for measuring and visualizing the impact of knowledge from the scientific literature [17], new techniques are being developed that have a better effect in inferring knowledge. With the confluence of recently advanced deep learning technologies, bibliometrics has been reborn as a new data mining technology with enhanced inferring ability to discover new knowledge from a latent knowledge base.

Knowledge graph, a graph-based machine-readable data structure, was originally developed to describe interactions between entities and has recently been used as a network-based knowledge discovery tool for understanding COVID-19 and finding a therapy for the disease [18,19,20,21].

Most existing studies extract the structure of the knowledge contained in accumulated databases. Therefore, for their results to become accurate, a significant quantity of data has to be accumulated. In this study, we try to determine a way to infer the characteristics of COVID-19 using the biomedical knowledge base accumulated so far without waiting for further knowledge to be significantly accumulated.

Word embedding techniques, one of the machine learning techniques, can extract knowledge by processing text and keywords, or obtain suggestions for new knowledge using relational reasoning and inference between keywords. This is because word embedding projects keywords onto space and expresses them as vectors [22]. Therefore, inference and analogy between keywords such as *France* − *Paris* = *Korea* − *Seoul*, or *France* − *Paris* + *Seoul* = *Korea* becomes possible mathematically. If we have information on France, Paris, and Seoul, it becomes possible to find Korea via word embedding. Using these characteristics, many studies on the use of word embedding are being conducted in each field. Word embedding is also widely used to understand biomedical entities [23].

When COVID-19 was first discovered, there was little knowledge about it, but studies on similar viruses, such as other coronaviruses and RNA viruses, have been accumulated. Using this knowledge to infer the characteristics of COVID-19, it may be possible to accelerate the discovery of solutions for COVID-19.

**Figure 1:**
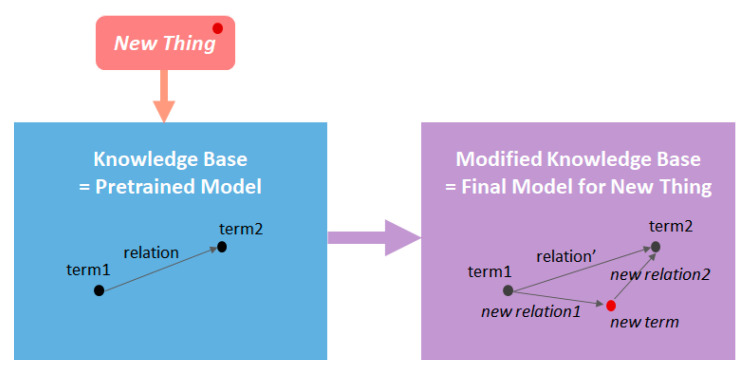
Conceptual diagram of research design.

In this study, we use word embedding and PubMed literature as the knowledge base (Figure 1). Over the past decades, a huge number of studies on viruses, drugs, proteins, and biological entities have been accumulated in PubMed. We try to apply inference of word embedding to the PubMed knowledge base to interpret the characteristics of COVID-19, even when knowledge of COVID-19 is still insufficient. For this, we strive to establish a knowledge base that fully represents the biomedical knowledge collection of the 2010s, that is, a balanced knowledge base, not biased towards a specific area. Then, a modified knowledge base is built by adding a small initial collection of early COVID-19-related articles (new thing). The knowledge base and the modified knowledge base are built into the pretrained model and final model through the word embedding technique. If the pretrained model expresses the knowledge base well, the modified knowledge base, inferring the relationship between the new term and pre-existing words, will be meaningful for understanding the characteristics of the new thing, i.e., COVID-19. To infer characteristics about COVID-19, we analyze the relationship between COVID-19 and two biomedical entities, namely drugs (chemicals), and proteins interacting with COVID-19. Where limited studies on COVID-19 and SARS-CoV-2 have been reported, we attempt to enhance our understanding of the virus using the existing knowledge stock on coronaviruses based on a modified knowledge base. This study aims to examine the potential of drug repurposing by applying word embedding to the PubMed literature. The relationship between COVID-19 and drugs as well as COVID-19 and proteins can then be deduced by the trained model.

## 2. Materials and Methods

### 2.1. Data: PubMed Literature

SARS-CoV-2 is a novel coronavirus, and the detrimental impact of the disease is too great to wait until enough research has been conducted to find a solution. Our aim is to determine information on SARS-CoV-2 using accumulated knowledge on known viruses, particularly coronaviruses, on PubMed, which is the largest and most updated literature database for research in the fields of biomedical and life sciences. The PubMed literature has a plethora of information on various subjects, which can be identified using the Medical Subject Headings (MeSH) and Substance Name (SN) of Unique Ingredient Identifiers (UNII), and the Chemical Abstracts Service (CAS) fields. MeSH and SN provide information sources that can be analyzed by extracting the subject keywords of publications. Using all the sentences included in the abstract of a given publication for the construction of the word embedding model, it would be possible to extract more keywords and relationships between keywords in text contents. However, if so, significant noise removal and keyword refinement are required, and this will take a long time. It is a matter of choice as to whether to secure a richer keyword dictionary or a refined keyword dictionary without noise, and we chose the latter for accurate inferring. To block data contamination fundamentally and to efficiently process and analyze data, we only used a controlled subject vocabulary from MeSH and SN. Regarding the PubMed literature pertaining to these two fields, we attempted to identify associations between COVID-19 and drugs as well as COVID-19 and proteins.

The analyzed dataset included 7,804,687 articles from PubMed published between 2010 and 2019; these articles were tagged with MeSH and SN terms. To infer the characteristics of COVID-19, all COVID-19-related articles published before 18 March 2020, were downloaded from PubMed. COVID-19-related articles that were not tagged with MeSH or SN terms were included using the Other Term (OT) field, which refers to the author keyword field. Unlike MeSH or SN terms, the OT category does not have a controlled vocabulary; thus, we further cleaned the terms. Keywords referring to COVID-19, such as “SARS-COV-19,” “2019 Novel Coronavirus,” and “Corona Virus disease 2019,” were all combined as COVID-19. The rest of the Other Terms were also appropriately refined. A total of 539 COVID-19-related articles were included in the analysis using OT.

#### 2.1.1. Medical Subject Headings (MeSH)

Each article on PubMed has several MeSH tags that represent the nature of the article’s subject. MeSH terms have 16 large categories: (a) Anatomy, (b) Organisms, (c) Diseases, (d) Chemicals and Drugs, (e) Analytical, Diagnostics and Therapeutic Techniques, and Equipment, (f) Psychiatry and Psychology, (g) Phenomena and Processes, (h) Disciplines and Occupations, (i) Anthropology, Education, Sociology, and Social Phenomena; (j) Technology, Industry, and Agriculture; (k) Humanities; (l) Information Science; (m) Named Groups; (n) Health Care; (o) Publication Characteristics; and (p) Geographical. Articles, each of which can have as few as one or as many as 40 or more tags. If two MeSH terms are tagged in the same article, the two MeSH terms are defined as being associated with one another. MeSH terms with a known pharmacological action are indexed as Pharmacological Action terms in the MeSH vocabulary system.

#### 2.1.2. Substance Name (SN)

When an article on PubMed literature mentions substances registered in the Unique Ingredient Identifier (UNII) and the Chemical Abstracts Service (CAS), the substance name becomes tagged in the Registry Number/EC Number and Substance Name fields. Registry Number and EC Number are codes registered in UNII and CAS, respectively, whereas Substance Name refers to the identification of the substance. Each article may have more than 20 substances tagged. If two substances are tagged in the same article, they are assumed to be associated with one another. Substance Names sometimes overlap with MeSH, but this rarely occurs. Of note, protein names are listed as broad terms in the MeSH vocabulary system, whereas they are listed in detail, along with the source, such as human, mouse, rat, or virus, in the Substance Name system.

#### 2.1.3. Vocabulary Combing MeSH, SN, and OT Terms

In the present study, a knowledge base was established using literature from PubMed. COVID-19-related articles were used to extract the relationship between COVID-19 and drugs and COVID-19 and proteins. The MeSH and SN terms, which efficiently express the subject of the article with little noise, were used to structure the knowledge base. For each article, the MeSH and SN terms were merged to create the combined vocabulary. The word-embedding model, a machine-learning technique, was then generated using co-occurrence relation information. To build the final model from the COVID-19-related article set, the OT terms of the COVID-19-related articles were added to expand the vocabulary further.

### 2.2. Model: Word Embedding with FastText

To broaden our understanding of COVID-19 and to infer new information about this disease, a new knowledge base needs to be established using existing knowledge bases. This study aims to produce a word-embedding model using an already established knowledge base, and to create a new knowledge base that allows the effective comparison and inference of the relationship between newly added information and the existing information. Knowledge base refers to the stock of knowledge that has been accumulated by researchers over the years. As COVID-19 is a novel issue, we aimed to build a knowledge base using the PubMed literature from the past ten years. Using the word-embedding model, every term within the knowledge base can be expressed as a vector; consequently, the relationship between terms can be calculated by vector computation.

Word embedding converts the sparse matrix that expresses relationships among numerous keywords (as the number of dimensions equals the number of keywords) into a dense matrix that condenses the number of dimensions (i.e., 100–200 dimensions). This allows the expression of keyword characteristics as vectors. All keywords within the vocabulary are expressed as vectors with appropriate dimensions, enabling the analysis of relationships among keywords using vector algebra. In addition, keyword analogy becomes possible, allowing a more efficient display of keyword relationships.

Common word-embedding models include Word2Vec [24] and FastText [25] for word-level embedding, and BERT (bidirectional encoder representations for transformers) [26] for sentence-level embedding. In this study, word-level embedding was used to embed biomedical terminology tagged in articles, such as MeSH and SN terms, with minimal noise and without requiring natural language processing or named entity recognition for sentences. We also tried to build our own pretrained and final models, considering the formation of an organic relationship between the two knowledge bases. Word2Vec and FastText employ very similar embedding methods; however, FastTex was selected for this study because it has a superior sub word-level analysis and out-of-vocabulary capabilities. Moreover, FastText can utilize the packages created by Facebook and Python-based Gensim. For this study, the Gensim package for FastText was used.

#### 2.2.1. Pretrained Model

FastText can use continuous-bag-of-words and skipgram models to infer relationships between words; in this study, the latter was used. MeSH and SN terms tagged in PubMed literature between 2010 and 2019 were used as data for FastText. The vocabulary consisted of 53,216 terms.

The three hyperparameters that have major impacts on the model characteristics in FastText (vector size, window size, and number of epochs) were tested for model optimization, whereas default values were used for other parameters. Vector size, which refers to the dimension of a word vector, was tested in 100, 150, and 200 settings. Window size, which describes the size of the context window used in measuring word pair relationships when building the word-embedding model, can go beyond 60 MeSH and SN terms per article. Therefore, window size was tested in 40, 50, and 60 settings. The number of epochs was tested in 10, 15, and 20 settings.

As the FastText model building in the present study was an unsupervised training, the following evaluation methods were applied for the model optimization test. First, the evaluate_word_pairs method provided by the Gensim package for FastText functions was utilized to perform plausibility validation of the medical term relation in the model. This method is similar to the one used by the National Center for Biotechnology Information (NCBI) of the US National Library of Medicine. According to [27], NCBI builds the word embedding model of PubMed and MeSH data using FastText; model evaluation is performed by measuring word pair similarity using Medical Resident Relatedness Set (UMNSRS) medical term pairs [28] from the University of Minnesota Pharmacy Informatics Lab. UMNSRS was developed by experts who manually evaluated the relatedness of 588 medical concept pairs. Out of these, the authors selected 145 pairs that were MeSH terms and used them for pretrained model evaluations. The evaluate_word_pairs method from Gensim calculates the Pearson correlation coefficient and the Spearman correlation coefficient between the FastText model and the list of UMNSRS medical term pairs. The model by [27] at NCBI showed a similarity of 0.660 to UMNSRS medical term pairs. The similarity to UMNSRS medical term pairs in this study had a Pearson correlation coefficient of above 0.667 and a Spearman correlation coefficient of above 0.663, as summarized in Table 1. Second, the country-capital pair list from Google’s question-answer.txt, which is a widely used list to evaluate word embedding of common words that appeared in PubMed literature, was also assessed. This method utilizes the analogy between word vectors in the word-embedding model and measures the agreement accuracy of the country–capital analogy relationship. As summarized in Table 1, the accuracy was above 0.785. Based on the two evaluations, the authors determined a vector size of 200, a window size of 50, and number of epochs of 10 as the optimal settings for the pretrained word-embedding model. A different model that exhibited higher accuracy in the second evaluation was considered; however, the results from the first evaluation were considered to be more relevant, as this is an embedding model for biomedical terms, and the Q-A accuracy of the model was found to be high (above 0.928).

#### 2.2.2. Final Model

The pretrained model was a word-embedding model using MeSH and SN terms from PubMed literature between 2010 and 2019. The final model was built by adding to the pretrained model the set of articles on COVID-19 published in 2020. As the number of COVID-19 articles tagged with MeSH and SN terms is not large, OT terms were used instead. The final model is a modified model, where a new thing, in other words, COVID-19, was added to the pretrained model; the root of this model was the same as that of the pretrained model. Therefore, vector size and window size among the three hyperparameters from the pretrained model were applied as fixed parameters in the final model. For the evaluation of the final model, only the number of epochs was used as a variable. Further, the final model evaluation requires a different method than the one used in the evaluation of the pretrained model. This is because the objectives of the two models are different. The pretrained model aims to build a knowledge base from the 2010s, whereas the final model aims to infer the characteristics of COVID-19. Word pair evaluation was applied to the pretrained model to structure the biomedical knowledge base using biomedical terms. In contrast, the final model needed to be evaluated to predict the characteristics of COVID-19 accurately using the pretrained model. However, in the early stage of research on COVID-19, there were not many publications on COVID-19; hence, a model that overfits only a very small part of what humanity has learned about COVID-19 would not be adequate. One solid basic knowledge about COVID-19 is that it is caused by RNA viruses. Therefore, we selected the most effective model based on the measured similarity of the COVID-19 term to RNA virus terms. As summarized in Table 2, the number of epochs ranged from 10 to 150, and the similarity between COVID-19 terms and RNA virus terms were measured. As the number of epochs increases, the learning is repeated, building a word embedding model that well describes the data of COVID-19 added to the final model, but at some point overfitting may occur, which hinders inferring about COVID-19. Therefore, we have to determine the appropriate number of epochs according to the evaluation method and build a final model. The average similarity increases as the number of epochs increases, reaches a maximum value at 110, and then tends to saturate somewhat. The highest average similarity was found with 110 epochs. Therefore, the final model used 110 epochs, and its vocabulary ultimately consisted of 53,316 terms, owing to the addition of the OT terms extracted from the COVID-19 article set to the pretrained model’s vocabulary.

## 3. Results

The following COVID-19-related drugs and proteins were extracted from the final model. From the list of drugs available, the authors focused on anti-infective drugs. For MeSH terms, Pharmacological Actions keywords are provided along with the drugs. The authors selected the following Pharmacological Action drugs to filter for anti-infective drugs: Anti-Bacterial Agents; Antibiotics, Antifungal; Antibiotics, Antineoplastic; Antibiotics, Antitubercular; Anti-Infective Agents; Anti-Infective Agents, Local; Anti-Infective Agents, Urinary; Antimalarials; Antiprotozoal Agents; Antitubercular Agents; Anti-HIV Agents; Antiviral Agents; HIV Fusion Inhibitors; HIV Integrase Inhibitors; and HIV Protease Inhibitors. Using these terms, a total of 401 anti-infective drugs emerged. Within the final model, the similarity between anti-infective drugs and COVID-19 was measured to assess for any relationship. Table 3 lists the top 100 out of the 401 anti-infective drugs that were related to the COVID-19 vaccine or to the treatment drugs currently being developed. The drugs in Table 3 that are highlighted in gray represent those that showed low relevance to COVID-19, compared with the top 100 drugs; however, these are currently being studied as potential vaccines or treatments. Excelra [29], the ReDO Project [30], and DrugBank [31] summarize the drugs that are being repurposed as potential COVID-19 vaccines or treatments. The authors compared these three drug repurposing databases and the final model results from the current study, and the comparison results are listed in Table 3, Reference column. Out of the 401 anti-infective drugs the authors selected, 64 drugs were identified to be in current development as COVID-19 vaccines or treatments. Based on the relevance to COVID-19, 33 repositioning candidate drugs were identified in the top 100 drugs. The imipenem and cilastatin drug combination (under the brand name Primaxin), which revealed the highest similarity, is a treatment for severe infections affecting the heart, lungs, bladder, kidney, skin, blood, bones, stomach, and the female reproductive organs. With the spread of COVID-19, the U.S. FDA approved the antibiotic combination of imipenem–cilastatin and relbactam (Recarbio) for the treatment of hospital-acquired bacterial pneumonia and ventilator associated bacterial pneumonia. Oseltamivir and chloroquine, the two drugs that were most frequently mentioned in the media in the first half of 2020, also showed a very high relevance to COVID-19. The amoxicillin and clavulanate potassium combination, more commonly known under the trade name Augmentin, is an antibiotic that is widely used for sinusitis, bronchitis, pneumonia, ear infections, and urinary tract and skin infections. Currently, clinical trials utilizing amoxicillin/clavulanate alone or in combination of azithromycin with amoxicillin/clavulanate are ongoing. The trimethoprim–sulfamethoxazole drug combination (Bactrim), which has excellent antibacterial activity against gram-negative bacteria and staphylococcus, is also an antibiotic used for the treatment of ear infections, urinary tract infections, bronchitis, traveler’s diarrhea, shigellosis, and *Pneumocystis jirovecii* pneumonia. The drug is currently in clinical trials for its use with Anakinra, an IL-1 receptor antagonist indicated for the treatment of the COVID-19-induced hyperimmune respiratory failure (aka cytokine storm).

Most of the potential drugs with the highest relevance (top 100) to COVID-19 were drugs for bacterial infections (antibiotics). Several drugs for viral infections were also on the list. Various anti-retrovirals (used in HIV/AIDS) and anti-malarial drugs were also shown to have high relevance to COVID-19.

To indirectly confirm the robustness of our final model, we compared the drug list of 10 models with different numbers of epochs. The top relevance drug list barely changed, and only the bottom relevance (about 10%) drug list showed small changes, indicating that our final model is robust and the list of potential drugs with the highest relevance (top 100) is a stable result.

Using a similar method, protein terms with high relevance to COVID-19 were extracted from the final model. Only the 5366 proteins that are of either human or coronavirus origin were extracted, and their relevance to COVID-19 was then analyzed. Table 4 lists the top 100 proteins relevant to COVID-19. The proteins highlighted in gray in Table 4 indicate those that showed low relevance to COVID-19 but are known to be human proteins that interact with COVID-19. Information on the human proteins that are known to interact with COVID-19 and on known proteins of COVID-19 can be found in [32] and in the study by [7]. Protein descriptions, gene names, and COVID-19 bait columns in Table 4 also lists the COVID-19 interacting proteins. In particular, COVID-19 viral proteins were identified as proteins with high relevance to COVID-19 in the final model, along with angiotensin converting enzyme 2, which is known as the COVID-19 entry receptor. Among the top 100 highly-relevant proteins, the following were identified: six SARS-CoV-2 viral proteins listed in The Human Protein Atlas (M protein, coronavirus; nsp1 protein, SARS coronavirus; nsp14 protein, SARS coronavirus; 3C-like proteinase, coronavirus; nonstructural protein 3, SARS coronavirus; Nsp16 protein, SARS virus) and three human proteins (angiotensin converting enzyme 2; NARS2 protein, human; ALG8 protein, human).

The drugs and proteins listed in Table 3 and Table 4 are the COVID-19 related term list extracted from PubMed MeSH and SN term-based word embedding model. When comparing these results with the latest references reflective of current research trends, some were consistent, while others highlighted information not being investigated in the current research.

## 4. Discussion and Conclusions

This research aimed to understand the characteristics of COVID-19, which is a novel disease that humanity is currently facing, using the PubMed database, a knowledge base that has been established over a long duration. To accomplish this, information from PubMed literature pertaining to coronaviruses from the past decade was structured in a word embedding model, and subsequently, the relationships between COVID-19 terms and other biomedical terms were inferred. With the result of this study, proteins and drugs with high relevance to COVID-19 were deduced.

The word embedding technique used in this study upgrades the field of knowledge discovery from the biomedical literature, previously dealt with in bibliometrics, enabling inference on the demand for knowledge with many uncertainties, such as that on COVID-19. This helps to understand and discover new knowledge. The vector calculation and mathematical modeling techniques of word embedding can play a role in advancing drug development, which is time-consuming and costly, by adding inferencing capabilities to the insufficient medical literature knowledge.

The result of this study is highly comparable to the biomedical demands of research and development efforts to overcome the COVID-19 crisis. We expect that this list of drugs and proteins, and their relevance to COVID-19, will help in identifying potential vaccine or treatment candidates. This word embedding research model also provided an in-silico drug design method for drug repurposing that can drastically reduce the time and cost of drug development. With the urgent need for identifying drug candidates for COVID-19, various data, tools and methods for drug repurposing are being introduced and analyzed. The results of this study also provide a computational method to predict potential drug-target interactions (DTIs).

This study exhibits three limitations. First, it only used MeSH and SN terms for word embedding, which both has advantages and limitations. As to the advantages, these terms are controlled vocabularies, and only technical terms were used to establish the model, which virtually eliminates all noise. However, it might have excluded new terms that may exist in plain texts. If plain texts such as abstracts would be included, natural language processing and named entity recognition could be required. In this case, the BERT model can be considered. Second, for drug repositioning, a broader consideration regarding the pharmacological action of drugs as anti-infective drugs should have been included. Recently, there have been cases of drugs being used for an entirely different indication; for example, anti-tumor drugs and anti-parasitic drugs are also being studied as potential COVID-19 treatments. As this study aims to expand our knowledge of COVID-19, it may also be necessary to observe more broadly its relevance to COVID-19. Third, adding more databases beyond PubMed can provide more information. In particular, adding clinical trials databases could be helpful in enriching the information by including data on the latest commercial drugs.

Follow-up research is needed to overcome these limitations. Future research should include the entire list of drug substance terms, as well as anti-infective drugs, for analysis in order to produce helpful results for drug repositioning for COVID-19. This is because, like the cases in which new indications were added for drugs with completely different indications in the past, it is not possible to rule out the possibility that a drug that appears to be irrelevant will appear as a therapeutic candidate for COVID-19. Furthermore, a word embedding model using clinical trial databases, in addition to PubMed literature, needs to be established. With the addition of pharmacokinetic prediction, the list of potential vaccine or treatment candidates could become more meaningful and more useful information. Studies to understand the interaction between drugs and proteins by applying a clustering technique to the drug list and protein list related to COVID-19, or studies applying the BERT model, are also meaningful as follow-up studies. If we approach the pandemic from the perspective of an X-event like a major accident [33], machine learning-based modeling studies of complex systems for the spread of infectious disease will also help broaden our understanding of COVID-19 and new infectious diseases caused by a novel virus [34]. These efforts will contribute to availing more accurate information pertaining to COVID-19 rapidly, which will help overcome new pandemics.

## Figures and Tables

**Table 1:** Word-embedding model evaluation results based on hyperparameter combinations.

No.	Hyperparameters			Evaluation_1				Evaluation_2
	Vector Size	Window Size	Number of Epochs	Pearson Correlation Similarity		Spearman Correlation Similarity		Q-A Accuracy
				Coefficient	*p*-Value	Coefficient	*p*-Value	
1	100	40	10	0.6774	2.13 × 10^−20^	0.6774	2.10 × 10^−20^	0.8810
2	100	40	15	0.6708	6.71 × 10^−20^	0.6776	2.02 × 10^−20^	0.8095
3	100	40	20	0.6687	9.62 × 10^−20^	0.6667	1.34 × 10^−19^	0.8095
4	100	50	10	0.6718	5.61 × 10^−20^	0.6759	2.77 × 10^−20^	0.8333
5	100	50	15	0.6634	2.34 × 10^−19^	0.6632	2.42 × 10^−19^	0.7857
6	100	50	20	0.6688	9.43 × 10^−20^	0.6713	6.10 × 10^−20^	0.8810
7	100	60	10	0.6747	3.41 × 10^−20^	0.6820	9.25 × 10^−21^	0.8810
8	100	60	15	0.6715	5.89 × 10^−20^	0.6736	4.10 × 10^−20^	0.8810
9	100	60	20	0.6745	3.51 × 10^−20^	0.6810	1.11 × 10^−20^	0.8571
10	150	40	10	0.6711	6.38 × 10^−20^	0.6816	9.99 × 10^−21^	0.9048
11	150	40	15	0.6749	3.29 × 10^−20^	0.6864	4.15 × 10^−21^	0.9762
12	150	40	20	0.6718	5.65 × 10^−20^	0.6802	1.29 × 10^−20^	0.9762
13	150	50	10	0.6775	2.07 × 10^−20^	0.6931	1.21 × 10^−21^	0.9286
14	150	50	15	0.6781	1.88 × 10^−20^	0.6914	1.66 × 10^−21^	0.9524
15	150	50	20	0.6772	2.19 × 10^−20^	0.6891	2.56 × 10^−21^	0.9762
16	150	60	10	0.6708	6.70 × 10^−20^	0.6817	9.79 × 10^−21^	0.9524
17	150	60	15	0.6780	1.89 × 10^−20^	0.6928	1.27 × 10^−21^	0.9762
18	150	60	20	0.6695	8.39 × 10^−20^	0.6796	1.42 × 10^−20^	0.9286
19	200	40	10	0.6714	6.05 × 10^−20^	0.6849	5.52 × 10^−21^	0.9524
20	200	40	15	0.6718	5.66 × 10^−20^	0.6856	4.81 × 10^−21^	0.9524
21	200	40	20	0.6700	7.66 × 10^−20^	0.6833	7.39 × 10^−21^	0.9048
22	200	50	10	0.6783	1.81 × 10^−20^	0.6936	1.09 × 10^−21^	0.9286
23	200	50	15	0.6761	2.64 × 10^−20^	0.6908	1.86 × 10^−21^	0.8810
24	200	50	20	0.6716	5.86 × 10^−20^	0.6843	6.13 × 10^−21^	0.9286
25	200	60	10	0.6708	6.65 × 10^−20^	0.6847	5.71 × 10^−21^	0.9524
26	200	60	15	0.6755	2.96 × 10^−20^	0.6875	3.39 × 10^−21^	1.0000
27	200	60	20	0.6669	1.30 × 10^−19^	0.6774	2.11 × 10^−20^	0.9524

**Table 2:** Final model evaluation results based on the number of epochs.

No.	Number of Epochs	Evaluation for Final Model
		COVID-19 with RNA Virus Term Similarity
1	10	0.4112
2	20	0.4524
3	30	0.4696
4	40	0.4779
5	50	0.4778
6	60	0.4802
7	70	0.4822
8	80	0.4835
9	90	0.4850
10	100	0.4862
11	110	0.4873
12	120	0.4867
13	130	0.4851
14	140	0.4853
15	150	0.4854

**Table 3:** List of drugs extracted from the final model with high relevance to COVID-19.

No	Drug Medical Subject Heading (MeSH) Terms for Anti-Infective PHARMACOLOGICAL Action from the Final Model	Original Indication	Reference
1	Cilastatin, Imipenem Drug Combination	Bacterial infection	
2	Oseltamivir	Influenza virus infection	PMID: 12690091, NCT04345419 et al., 7 cases
3	Chloroquine	Malaria	PMID: 32074550, NCT04286503 et al., 29 cases
4	Amoxicillin-Potassium Clavulanate Combination	Bacterial infection	NCT04363060
5	Trimethoprim, Sulfamethoxazole Drug Combination	Bacterial infection	NCT04357366, NCT03489629
6	Emtricitabine, Rilpivirine, Tenofovir Drug Combination	HIV/AIDS	
7	Colistin	Bacterial infection	ChiCTR2000032242 (China)
8	Interferons	Viral infection, Cancer	NCT04379518 et al., 9 cases
9	Artemether, Lumefantrine Drug Combination	Malaria	
10	Penicillin	Bacterial infection	
11	Bacteriocins	Bacterial infection	
12	Amdinocillin	Bacterial infection	
13	Tigecycline	Bacterial infection	PMID: 28700943
14	Streptogramins	Bacterial infection	
15	Teicoplanin	Bacterial infection	IRCT20161204031229N3 (Iran)
16	Palivizumab	Viral infection	
17	Aztreonam	Bacterial infection	
18	Meropenem	Bacterial infection	
19	Azlocillin	Bacterial infection	
20	Silver Proteins	Antiseptics	
21	Imipenem	Bacterial infection	
22	Ribavirin	Viral infection	PMID: 22555152, NCT04392427
23	Lincosamides	Bacterial infection	
24	Piperacillin, Tazobactam Drug Combination	Bacterial infection	NCT02735707
25	Polymyxins	Bacterial infection	
26	Emtricitabine, Tenofovir Disoproxil Fumarate Drug Combination	HIV/AIDS	NCT04329520
27	Mefloquine	Malaria	NCT04347031
28	Methicillin	Bacterial infection	
29	Zanamivir	Influenza A virus infection	PMID: 15200845
30	Rimantadine	Influenza A virus infection	PMID: 31133031, 15288617
31	Valganciclovir	Viral infection	
32	Amantadine	Dyskinesia associated with parkinsonism, influenza infection	
33	Cephalosporins	Bacterial infection	
34	Ampicillin	Bacterial infection	
35	Doripenem	Bacterial infection	
36	Simeprevir	HCV infection	
37	Lopinavir	HIV/AIDS	NCT04372628 et al., 37 cases
38	Cefamandole	Bacterial infection	
39	Ceftriaxone	Bacterial infection	NCT02735707
40	Thienamycins	Bacterial infection	
41	Penicillic Acid	Bacterial infection	
42	Sisomicin	Bacterial infection	
43	Ganciclovir	Cytomegalovirus retinitis	PMID: 32166607
44	Primaquine	Malaria	NCT04349410
45	Sulfalene	Bacterial infection	
46	Azithromycin	Bacterial infection	NCT04332107 et al. 67 cases
47	Vancomycin	Bacterial infection	NCT02667418
48	Spectinomycin	Bacterial infection	
49	Efavirenz, Emtricitabine, Tenofovir Disoproxil Fumarate Drug Combination	HIV/AIDS	
50	Minocycline	Bacterial infection	NCT03489629
51	Leucomycins	Bacterial infection	
52	Ticarcillin	Bacterial infection	
53	Linezolid	Bacterial infection	PMID: 16127068, 16723564, 22094260
54	Ertapenem	Bacterial infection	
55	Clindamycin	Bacterial infection	NCT04349410
56	Chloramphenicol	Bacterial infection	PMID: 23148581
57	Doxycycline	Bacterial infection	NCT04370782 et al., 6 cases
58	Hydroxychloroquine	Malaria	NCT04358068 et al., 177 cases
59	Famciclovir	viral infection	
60	Tyrocidine	Bacterial infection	
61	Acyclovir	viral infection	
62	Nisin	Bacterial infection	
63	Nebramycin	Bacterial infection	
64	Penicillanic Acid	Bacterial infection	
65	Elvitegravir, Cobicistat, Emtricitabine, Tenofovir Disoproxil Fumarate Drug Combination	HIV/AIDS	
66	Pristinamycin	Bacterial infection	
67	Nevirapine	HIV/AIDS	
68	Lamivudine	HIV/AIDS	
69	Piperacillin	Bacterial infection	NCT04394182
70	Valacyclovir	viral infection	
71	Viomycin	Bacterial infection	
72	Emtricitabine	HIV/AIDS	NCT04334928
73	Ceftazidime	Bacterial infection	
74	Artemisinins	Malaria	
75	Josamycin	Bacterial infection	
76	Telbivudine	viral infection	
77	Fidaxomicin	Bacterial infection	NCT02667418
78	Edeine	Bacterial infection	
79	Cefoxitin	Bacterial infection	
80	Proguanil	Malaria	
81	Fosfomycin	Bacterial infection	
82	Metha-cycline	Bacterial infection	
83	Tylosin	Bacterial infection	
84	Sulbactam	Bacterial infection	
85	Amikacin	Bacterial infection	
86	Ritonavir	HIV/AIDS	NCT04372628 et al., 43 cases
87	Sulfa-doxine	Malaria	
88	Dihydrostreptomycin Sulfate	Bacterial infection	
89	Cefotaxime	Bacterial infection	
90	Cefotetan	Bacterial infection	
91	Hexetidine	Bacterial infection	
92	Atovaquone	Pneumocystis pneumonia, toxoplasmosis, malaria	NCT04339426
93	Oxacillin	Bacterial infection	
94	Daptomycin	Bacterial infection	
95	Rilpivirine	HIV/AIDS	
96	Sofosbuvir	Hepatitis C virus infection	NCT04443725
97	Streptomycin	Bacterial infection	
98	Artesunate	Malaria	NCT04387240
99	Hepcidins	Antimicrobial peptide	
100	Sparsomycin	Bacterial infection	
108	Tenofovir	Viral infection	IRCT20200421047155N1
134	Mupirocin	Impetigo and secondary skin infection	NCT03489629
137	Inosine Pranobex	Viral infection	NCT04360122, NCT04383717
142	Cytarabine	Leukemia	NCT02310321
149	Clarithromycin	Bacterial infection	NCT04398004
150	Itraconazole	Fungal infection	2020-001243-15 (Begium)
154	Amoxicillin	Bacterial infection	NCT04363060
157	Tazobactam	Bacterial infection	NCT04394182
160	Cobicistat	HIV-1 infection	NCT04425382 et al., 3 cases
175	Quinacrine	Malaria	PMID: 23301007, 31307979, 32194980
186	Darunavir	HIV-1 infection	NCT04435587 et al., 4 cases
191	Iodine	Breast disorders and pain	NCT04344236
194	Indinavir	HIV/AIDS	PMID: 15144898
199	Clavulanic Acid	Bacterial infection	NCT04363060
202	Mycophenolic Acid	Organ rejection	PMID: 5799033
204	Maraviroc	HIV infection	NCT04435522, NCT04441385
220	Chlorhexidine	Antiseptics	NCT04344236, NCT03489629
235	Trimethoprim	Bacterial infection	NCT04357366, NCT03489629
247	Sulfamethoxazole	Bacterial infection	NCT04357366, NCT03489629
253	Acetylcysteine	Mucolytics	NCT04419025 et al. 4 cases
257	Dactinomycin	Cancer	PMID: 1335030, 32194980
269	Atazanavir Sulfate	HIV-1 infection	NCT02016924
277	Idarubicin	Acute Myeloid Leukemia	NCT02310321
284	Hydrogen Peroxide	Disinfectant and Sterilizer	NCT04409873
291	Povidone-Iodine	Infection	NCT04410159 et al., 7 cases
294	Sirolimus	Organ rejection	NCT04374903 et al., 3 cases
298	Methylene Blue	Methemoglobinemia	NCT04376788, NCT04370288
350	Pyrazinamide	Tuberculosis	NCT04349241
362	Camphor	Coughing	PMID: 27823881, 32194980
367	Cetylpyridinium	Bacterial infection	NCT04409873
374	Daunorubicin	Cancer	PMID: 9647783

**Table 4:** List of coronavirus or human proteins extracted from the final model with high relevance to COVID-19 with protein description, gene name and covid-19 bait information.

No	Protein Substance Name (SN) Terms of Human and Coronavirus from the Final Model	Protein Description	Gene Name	Covid-19 Bait
1	M protein, Coronavirus	SARS-CoV-2 Viral Protein (M)		
2	Nsp1 protein, SARS coronavirus	SARS-CoV-2 Viral Protein (nsp1)		
3	nsp14 protein, SARS coronavirus	SARS-CoV-2 Viral Protein (nsp14)		
4	3C-like proteinase, Coronavirus	SARS-CoV-2 Viral Protein (nsp5)		
5	dynorphin converting enzyme			
6	COG2 protein, human			
7	nonstructural protein 3, SARS coronavirus	SARS-CoV-2 Viral Protein (nsp3)		
8	angiotensin converting enzyme 2	SARS-CoV-2 entry receptors	ACE2	
9	COX6A1 protein, human			
10	poly U polymerase			
11	CORIN protein, human			
12	COX8C protein, human			
13	Nsp16 protein, SARS virus	SARS-CoV-2 Viral Protein (nsp16)		
14	COX5A protein, human			
15	COQ5 protein, human			
16	GBE1 protein, human			
17	transmembrane serine protease 2, human			
18	sfericase			
19	CPVL protein, human			
20	COX4I1 protein, human			
21	LARS2 protein, human			
22	COX5B protein, human			
23	NARS2 protein, human	SARS-CoV-2 interacting protein	NARS2	SARS-CoV-2 nsp8
24	UL49A protein, Human herpesvirus 2			
25	COX6B1 protein, human			
26	PARS2 protein, human			
27	hydrogenase maturating endopeptidase HYBD			
28	VARS2 protein, human			
29	human airway trypsin-like protease			
30	ERI1 protein, human			
31	Myxo-bacter alpha-lytic proteinase			
32	AARS2 protein, human			
33	RARS2 protein, human			
34	Tli polymerase			
35	ADAM29 protein, human			
36	HPN protein, human			
37	O-antigen polymerase			
38	SPEG protein, human			
39	CLPB protein, human			
40	FONG protein, human			
41	ERManI protein, human			
42	PDIK1L protein, human			
43	ALG8 protein, human	SARS-CoV-2 interacting protein	ALG8	SARS-CoV-2 orf9c
44	NVL protein, human			
45	HFM1 protein, human			
46	HARS2 protein, human			
47	COASY protein, human			
48	TMPRSS13 protein, human			
49	C1RL protein, human			
50	COX20 protein, human			
51	ECEL1 protein, human			
52	NARFL protein, human			
53	GANAB protein, human			
54	AFG3L2 protein, human			
55	TSEN54 protein, human			
56	ERAL1 protein, human			
57	m-AAA proteases			
58	KY protein, human			
59	TMEM129 protein, human			
60	KEL protein, human			
61	APH1B protein, human			
62	MGME1 protein, human			
63	ATL3 protein, human			
64	oxacillinase			
65	COX10 protein, human			
66	MYORG protein, human			
67	hemagglutinin-protease			
68	Tiki1 protein, human			
69	FIGN protein, human			
70	ATL1 protein, human			
71	RLGP protein, human			
72	FbxL4 protein, human			
73	hemorrhagic metalloproteinase			
74	3C proteases			
75	HEXB protein, human			
76	GNPTG protein, human			
77	ADAM23 protein, human			
78	NSF protein, human			
79	RNA polymerase SP6			
80	ADAM22 protein, human			
81	IntS9 protein, human			
82	SERAC1 protein, human			
83	RPL41 protein, human			
84	pokeweed antiviral protein			
85	COX15 protein, human			
86	small cardioactive peptide A			
87	DARS2 protein, human			
88	AGBL5 protein, human			
89	LARGE1 protein, human			
90	COX4I2 protein, human			
91	NHLH1 protein, human			
92	MINDY2 protein, human			
93	DHX29 protein, human			
94	RNA polymerase Esigma(38)			
95	ADAM30 protein, human			
96	DLG2 protein, human			
97	Ric-8b protein, human			
98	UST protein, human			
99	Deep Vent DNA polymerase			
100	PIGL protein, human			
143	EXOSC8 protein, human	SARS-CoV-2 interacting protein	EXOSC8	SARS-CoV-2 nsp8
163	PITRM1 protein, human	SARS-CoV-2 interacting protein	PITRM1	SARS-CoV-2 M
172	NGLY1 protein, human	SARS-CoV-2 interacting protein	NGLY1	SARS-CoV-2 orf8
177	ALG11 protein, human	SARS-CoV-2 interacting protein	ALG11	SARS-CoV-2 nsp4
281	TMPRSS2 protein, human	SARS-CoV-2 entry associated proteases	TMPRSS2	
345	PCSK6 protein, human	SARS-CoV-2 interacting protein	PCSK6	SARS-CoV-2 orf8
352	MDN1 protein, human	SARS-CoV-2 interacting protein	MDN1	SARS-CoV-2 orf7a
360	ERMP1 protein, human	SARS-CoV-2 interacting protein	ERMP1	SARS-CoV-2 orf9c
384	QSOX2 protein, human	SARS-CoV-2 interacting protein	QSOX2	SARS-CoV-2 nsp7
392	HectD1 protein, human	SARS-CoV-2 interacting protein	HECTD1	SARS-CoV-2 nsp8
497	USP54 protein, human	SARS-CoV-2 interacting protein	USP54	SARS-CoV-2 nsp12
502	NDUFB9 protein, human	SARS-CoV-2 interacting protein	NDUFB9	SARS-CoV-2 orf9c
577	NEU1 protein, human	SARS-CoV-2 interacting protein	NEU1	SARS-CoV-2 orf8
664	PRIM1 protein, human	SARS-CoV-2 interacting protein	PRIM1	SARS-CoV-2 nsp1
685	Cwc27 protein, human	SARS-CoV-2 interacting protein	CWC27	SARS-CoV-2 E
691	NDUFAF1 protein, human	SARS-CoV-2 interacting protein	NDUFAF1	SARS-CoV-2 orf9c
696	AASS protein, human	SARS-CoV-2 interacting protein	AASS	SARS-CoV-2 M
716	FKBP10 protein, human	SARS-CoV-2 interacting protein	FKBP10	SARS-CoV-2 orf8
740	ATP6V1A protein, human	SARS-CoV-2 interacting protein	ATP6V1A	SARS-CoV-2 M
756	Mov10 protein, human	SARS-CoV-2 interacting protein	MOV10	SARS-CoV-2 N
773	TCF12 protein, human	SARS-CoV-2 interacting protein	TCF12	SARS-CoV-2 nsp12
785	TBK1 protein, human	SARS-CoV-2 interacting protein	TBK1	SARS-CoV-2 nsp13
935	DDX21 protein, human	SARS-CoV-2 interacting protein	DDX21	SARS-CoV-2 N
938	DDX10 protein, human	SARS-CoV-2 interacting protein	DDX10	SARS-CoV-2 nsp8
1010	UPF1 protein, human	SARS-CoV-2 interacting protein	UPF1	SARS-CoV-2 N
1026	ACAD9 protein, human	SARS-CoV-2 interacting protein	ACAD9	SARS-CoV-2 orf9c
1080	ADAMTS1 protein, human	SARS-CoV-2 interacting protein	ADAMTS1	SARS-CoV-2 orf8
1118	GFER protein, human	SARS-CoV-2 interacting protein	GFER	SARS-CoV-2 nsp10
1120	RNF41 protein, human	SARS-CoV-2 interacting protein	RNF41	SARS-CoV-2 nsp15
1145	ADAM9 protein, human	SARS-CoV-2 interacting protein	ADAM9	SARS-CoV-2 orf8
1217	PPT1 protein, human	SARS-CoV-2 interacting protein	PPT1	SARS-CoV-2 orf10
1300	LOX protein, human	SARS-CoV-2 interacting protein	LOX	SARS-CoV-2 orf8
1450	MYCBP2 protein, human	SARS-CoV-2 interacting protein	MYCBP2	SARS-CoV-2 nsp12
1481	CTSL protein, human	SARS-CoV-2 entry associated proteases	CTSL	
1483	CYB5R3 protein, human	SARS-CoV-2 interacting protein	CYB5R3	SARS-CoV-2 nsp7
1621	NEK9 protein, human	SARS-CoV-2 interacting protein	NEK9	SARS-CoV-2 nsp9
1693	COMT protein, human	SARS-CoV-2 interacting protein	COMT	SARS-CoV-2 nsp7
1709	MARK3 protein, human	SARS-CoV-2 interacting protein	MARK3	SARS-CoV-2 orf9b
1798	HS6ST2 protein, human	SARS-CoV-2 interacting protein	HS6ST2	SARS-CoV-2 orf8
1816	MARK2 protein, human	SARS-CoV-2 interacting protein	MARK2	SARS-CoV-2 orf9b
1840	Rab14 protein, human	SARS-CoV-2 interacting protein	RAB14	SARS-CoV-2 nsp7
1845	G3BP1 protein, human	SARS-CoV-2 interacting protein	G3BP1	SARS-CoV-2 N
1871	Rab10 protein, human	SARS-CoV-2 interacting protein	RAB10	SARS-CoV-2 nsp7
1957	MARK1 protein, human	SARS-CoV-2 interacting protein	MARK1	SARS-CoV-2 orf9b
2109	RAB8A protein, human	SARS-CoV-2 interacting protein	RAB8A	SARS-CoV-2 nsp7
2279	USP13 protein, human	SARS-CoV-2 interacting protein	USP13	SARS-CoV-2 nsp13
2287	RAB5C protein, human	SARS-CoV-2 interacting protein	RAB5C	SARS-CoV-2 nsp7
2346	PRKACA protein, human	SARS-CoV-2 interacting protein	PRKACA	SARS-CoV-2 nsp13
2369	PLAT protein, human	SARS-CoV-2 interacting protein	PLAT	SARS-CoV-2 orf8
2436	PTGES2 protein, human	SARS-CoV-2 interacting protein	PTGES2	SARS-CoV-2 nsp7
2598	BRD2 protein, human	SARS-CoV-2 interacting protein	BRD2	SARS-CoV2 E
2722	PLOD2 protein, human	SARS-CoV-2 interacting protein	PLOD2	SARS-CoV-2 orf8
2744	RALA protein, human	SARS-CoV-2 interacting protein	RALA	SARS-CoV-2 nsp7
2847	DPP4 protein, human	SARS-CoV-2 entry receptors	DPP4	
3039	NSD2 protein, human	SARS-CoV-2 interacting protein	NSD2	SARS-CoV-2 nsp8
3204	MIB1 ligase, human	SARS-CoV-2 interacting protein	MIB1	SARS-CoV-2 nsp9
3456	CTSB protein, human	SARS-CoV-2 entry associated proteases	CTSB	
4470	RHOA protein, human	SARS-CoV-2 interacting protein	RHOA	SARS-CoV-2 nsp7
4471	SIRT5 protein, human	SARS-CoV-2 interacting protein	SIRT5	SARS-CoV-2 nsp14
4540	DNMT1 protein, human	SARS-CoV-2 interacting protein	DNMT1	SARS-CoV-2 orf8
4569	HMOX1 protein, human	SARS-CoV-2 interacting protein	HMOX1	SARS-CoV-2 orf3a
4663	IMPDH2 protein, human	SARS-CoV-2 interacting protein	IMPDH2	SARS-CoV-2 nsp14
4780	RIPK1 protein, human	SARS-CoV-2 interacting protein	RIPK1	SARS-CoV-2 nsp12
4929	HDAC2 protein, human	SARS-CoV-2 interacting protein	HDAC2	SARS-CoV-2 nsp5

## Data Availability

The pretrained and the final models in this study are obtained from https://github.com/hyyangkisti/bio_embedding. The whole lists of drugs and proteins related COVID-19 are available at https://hyyangkisti.github.io/ddppr/covid-19. (These sites were accessed on 15 March 2021).
